# On the Treatment of Puerperal Peritonitis by Large Doses of Opium

**Published:** 1855-03

**Authors:** A. Clark

**Affiliations:** Professor of Physiology and Pathology in the College of Physicians and Surgeons, N. Y.


On the treatment of Puerperal Peritonitis by large doses of Opium. By A. Clark M. D., Professor of Physiology and Pathology in the College of Physicians and Surgeons, N. Y. Professor Clark has communicated to the new American edition of *Ramsbotham's System of Obstretrics*, his experience in the treatment of puerperal fever, by opium, at Bellevue Hospital, N. Y. Following the relation of one case and a reference to several others, he concludes:

In reviewing the cases, of which an outline is given above, I think the following conclusions are justifiable:

1st. When a prominent element in "puerperal fever" is peritonitis, the treatment with large doses of opium is more successful than any other that has yet been proposed.

2d. To be successful, this treatment must be commenced early, and the patient must be brought under its influence as rapidly as the susceptibility of the system can be ascertained by trial.

3d. The quantity of opium required to produce a safe but desirable degree of narcotinism, varies greatly in different cases; so that it is necessary to begin with doses that cannot do mischief, and increase every two hours till the influence of the opiate is sufficiently decided.

4th. Every dose, during at least the whole tenative period, should be administered by the physician himself, or by some person on whose knowledge of the effects of opium and whose watchfulness and discretion he can rely. Some young physicians are too bold, and endanger the life of the patient; others are too timid, and do not control the disease.

5th. The opium treatment alone will not cure "puerperal fever," when its leading element is purulent metritis, though there is reason to believe that it will control, and even prevent the peritonitis which generally accompanies it. This conclusion has been confirmed by recent observations.

6th. The tolerance of opium in some cases of puerperal peritonitis almost surpasses belief. Yet in private practice I have not found more than half or two-thirds of a grain of sulph. morph., every two hours, necessary, and have generally began with less, except for the first dose.

7th. The influence of the opium should be kept up till the pain and tenderness subside, the tympanites diminishes in some degree, and the pulse falls below 100—then, with the concurrence of other symptoms, it should be gradually diminished, and at length discontinued.

A few remarks and statements may be needed to make some of these conclusions intelligible.

The usual effects of opium given in efficient doses for the cure of this disease, are, a disposition to sleep, but not profoundly; a contracted pupil; perspiration, often profuse; sometimes a red, blotchy eruption; diminished frequency of the respiration; subsidence of pain and tenderness; slight suffusion of the eyes; and after a variable time, reduced frequency of pulse. Of these effects, three have been chiefly regarded as the criterion by which each particular dose is to be governed. If, when a dose is due, the sleep is profound (the amount of sleep is of little importance, if the patient be easily roused from it), there is reason to hesitate; if the respiration has been already reduced to twelve in the minute, and is *very* irregular and sighing, the dose should be diminished or wholly withheld; yet so long as the tenderness continues, it is desirable to urge the opiate, but, of course, always within the limits of safety.

The respiration appears to be the most certain indication of danger. I have not generally aimed to reduce it below 12 the minute. Yet in almost every case it has fallen, once or twice in the course of the treatment, as low as 7, and sometimes to 5. In no instance, however, has the narcotism, taken as a whole, been so profound as in the case detailed above. No instance of fatal narcotism has yet occurred under my observation, nor among the many cases reported to me by others.

Regarding the tolerance of opiates in some of these cases—at the risk of being charged with rashness and trifling with human life, I will make some extracts from case 7. The treatment was commenced at 10 A. M., on the 26th of December—two grains of opium hourly. At 2 P. M., no change in symptoms, dose increased to gr. iv. At 3, gr. iv. At 4, gr. v. At 5, gr. v. At 6, gr. viij. At 8, gr. x. At 9, gr. xij. At 11, sol. morph. sulph. (sixteen gr. to f ʒi) ʒiss. At 12, ʒj. At 1½ A. M. (respiration 6), 0. At 6 A. M. (respiration 12), opium gr. xij. At 10, sol. ʒj. At 12 M., opium gr. xij. At 1½ P. M., sol. ʒii. At 2½, ʒii. At 3½, opium xxiv. At 5, gr. xij. At 6½, sol. ʒijss. At 7½, ʒij. At 9, opium gr. xiv. At 10, gr. xvj. At 11, gr. xviij. 28th, at 1 A. M., sol. ʒiiss. At 2, ʒiv. At 3¼, opium gr. xx. At 4, sol. ʒiiss. At 5, ʒiij. At 6, ʒiiiss. At 6½, opium gr. x. At 7, sol. ʒiiiss. At 8, opium gr. xxij. At 8½, sol. ʒiv. At 10, ʒiij. At 11½, ʒiij. At 12, 0. Thus this woman took, in the first 26 hours of her treatment, opium gr. lxviij., and sulph. morph. gr. vij.; or, counting one grain of sulph. morph. as four grains of opium, one hundred and six (106) grains of opium. In the second 24 hours, she took opium gr. cxlviij., and sulph. morph. gr. lxxxj., or opium four hundred and seventy two (472) grains. On the third day, she took 236 grains. On the fourth, 120 grains. On the fifth, 54 grains. On the sixth, 22 grains. On the seventh day, 8 grains. After which, the treatment was wholly suspended. This woman was not addicted to drinking, and after her recovery, she assured me repeatedly that she did not know opium by sight, and had never taken it, or any of its preparations, unless it had been prescribed by a physician. This is, perhaps, "horrible dosing," and only justifiable as an experiment on a desperate disease. Yet this woman is alive to tell her story, as are several others who took surprising quantities of this drug. But later observations have shown that the tenth to the twentieth part of this maximum is efficient in controlling the disease. So this case is referred to, not for imitation, but because, with similar cases, it is a medical curiosity; and may, perhaps, open some new therapeutical views.

The result of the opium treatment in the hands of my professional friends in this city have not been uniformly successful. This was to have been expected. When the path to success is so narrow, and so little trodden, though beset with dangers on both sides, it is unavoidable that many will lose it. But I believe I am authorised in saying, that those who have seen most of this mode of medication, are most attached to it. It is not to be expected that in a disease so dangerous as the one under consideration, any plan can be uniformly successful, even with advantages of accurate diagnosis and early treatment; but, when it is remembered that the diagnosis between purulent metritis and puerperal peritonitis is not always easy,—and that this medication is successful in proportion to its early adoption,—we may probably find reason for its failure in other hands, as well as in my own.

By way of illustrating the vigilance and discretion which must be exercised in the administration of each successive dose of the opiate in this mode of treatment, I will add, that it could never have been fairly tested by me without the zealous, intelligent, and untiring assistance of the house-physicians of the Hospital. They visited the patients every hour by night as well as by day, and every dose of the medicine, from the first case to the last, was given by them, and proportioned to the hourly exigencies.—*New York Journal of Medicine.*

